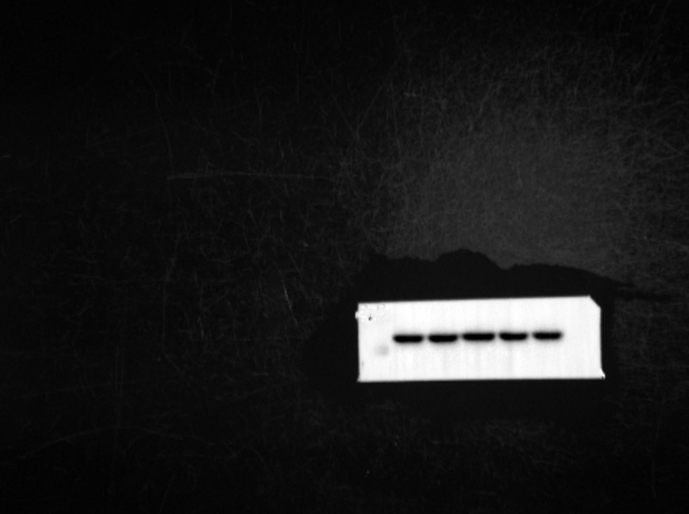# Dimethyl fumarate ameliorates oxidative stress-induced acute kidney injury after traumatic brain injury by activating Keap1-Nrf2/HO-1 signaling pathway

**DOI:** 10.1016/j.heliyon.2024.e32377

**Published:** 2024-06-04

**Authors:** Mei-zhu Gao, Jing-yi Zeng, Xue-jing Chen, Lan Shi, Fu-yuan Hong, Miao Lin, Jie-wei Luo, Han Chen

**Affiliations:** a Department of Nephrology, Shengli Clinical Medical College of Fujian Medical University, Fujian Provincial Hospital, Fuzhou, Fujian, 350001, China; b Department of Intensive Care Medicine, Shengli Clinical Medical College of Fujian Medical University, Fujian Provincial Hospital, Fuzhou, Fujian, 350001, China; c Department of Traditional Chinese Medicine, Shengli Clinical Medical College of Fujian Medical University, Fujian Provincial Hospital, Fuzhou, Fujian, 350001, China; d The Fourth Department of Critical Care Medicine, Shengli Clinical Medical College of Fujian Medical University, Fujian Provincial Hospital, Fujian Provincial Center for Critical Care Medicine, Fujian Provincial Key Laboratory of Critical Care Medicine, Fuzhou, Fujian, 350001, China

**Keywords:** Acute kidney injury, Traumatic brain injury, Oxidative stress, Keap1-Nrf2/HO-1 signaling pathway, Dimethyl fumarate

## Abstract

Acute kidney injury (AKI) frequently emerges as a consequential non-neurological sequel to traumatic brain injury (TBI), significantly contributing to heightened mortality risks. The intricate interplay of oxidative stress in the pathophysiology of TBI underscores the centrality of the Keap1-Nrf2/HO-1 signaling pathway as a pivotal regulator in this context. This study endeavors to elucidate the involvement of the Keap1-Nrf2/HO-1 pathway in modulating oxidative stress in AKI subsequent to TBI and concurrently explore the therapeutic efficacy of dimethyl fumarate (DMF). A rat model of TBI was established via the Feeney free-fall method, incorporating interventions with varying concentrations of DMF. Assessment of renal function ensued through measurements of serum creatinine and neutrophil gelatinase-associated lipocalin. Morphological evaluation of renal pathology was conducted employing quantitative hematoxylin and eosin staining. The inflammatory response was scrutinized by quantifying interleukin (IL)-6, IL-1β, and tumor necrosis factor-α levels. Oxidative stress levels were discerned through quantification of malondialdehyde and superoxide dismutase. The apoptotic cascade was examined via the terminal deoxynucleotidyl transferase dUTP deletion labeling assay. Western blotting provided insights into the expression dynamics of proteins affiliated with the Keap1-Nrf2/HO-1 pathway and apoptosis. The findings revealed severe kidney injury, heightened oxidative stress, inflammation, and apoptosis in the traumatic brain injury model. Treatment with DMF effectively reversed these changes, alleviating oxidative stress by activating the Keap1-Nrf2/HO-1 signaling pathway, ultimately conferring protection against AKI. Activating Keap1-Nrf2/HO-1 signaling pathway may be a potential therapeutic strategy for attenuating oxidative stress-induced AKI after TBI.

## 1 Introduction

Traumatic brain injury (TBI) is a severe and rapidly progressing condition characterized by high mortality and disability rates [1]. In addition to the primary injury, TBI can lead to various secondary complications, including acute kidney injury (AKI) [[2], [3], [4]]. AKI, with a reported incidence ranging from 11.9 % to 26.7 % in traumatic brain injury cases, stands as a frequent and significant non-neurological complication associated with increased mortality [2]. Unraveling the pathogenesis of post-traumatic secondary injury and developing targeted therapeutic interventions hold paramount clinical significance. TBI can result in cerebral ischemia and hypoxia, leading to disturbed cellular metabolism and energy depletion [[5], [6], [7]]. This disruption in cellular energy supply triggers the release of excitatory amino acids and the generation of free radicals, causing oxidative stress [8,9]. The attack of free radicals on biological macromolecules such as lipids, proteins, and nucleic acids aggravates the damage of brain tissue cells and mitochondrial membranes, triggering a series of pathological processes including inflammation and apoptosis [10,11]. TBI also directly induces the production of pro-inflammatory cytokines to activate the inflammatory response, which not only damages the organ causing cell death, but also destroys antioxidant defenses, thus exacerbating the vicious cycle. Similarly, oxidative stress, induced by various pathological conditions such as ischemia-reperfusion, intoxication and contrast agents, acts as a critical mediator in the development and progression of AKI, affecting various cellular processes and pathways involved in renal injury [12,13]. In other words, oxidative stress, inflammation, and apoptosis together contribute to the development of both AKI and TBI.

The Keap1-Nrf2/HO-1 signaling pathway is a key cellular defense mechanism against oxidative stress. Under normal conditions, Nrf2 is bound to its negative regulator, Keap1, in the cytoplasm, leading to the inhibition of Nrf2 activity. However, under stress conditions, Keap1 undergoes conformational changes, releasing Nrf2 and allowing it to translocate into the nucleus. In the nucleus, Nrf2 binds to antioxidant response elements (AREs) and activates the expression of various downstream antioxidant genes, including hemeoxygenase-1 (HO-1) [14]. Activated Nrf2 can bind to the gene regions of Bax and Bcl proteins, thereby modulating their expression. Specifically, Nrf2 activation upregulates the expression of the anti-apoptotic protein Bcl-2 while concurrently inhibiting the expression of the pro-apoptotic protein Bax.

However, it is unclear whether the Keap1-Nrf2/HO-1 signaling pathway is involved in the development of AKI after TBI.

We hypothesize that activation of the Keap1-Nrf2/HO-1 signaling pathway can attenuate oxidative stress and ameliorate AKI in TBI. In this study, we evaluated the severity of secondary AKI in a rat model of TBI and investigated the levels of inflammation and oxidative stress in renal and brain tissues. Furthermore, we aimed to explore the role of Keap1-Nrf2/HO-1 pathway activation in regulating these symptoms. We anticipated that dimethyl fumarate (DMF), an agonist of Nrf2 that activates the Keap1-Nrf2/HO-1 pathway, would alleviate oxidative stress and improve AKI in TBI.

## 2 Methods

### 2.1 Animals

Male Sprague Dawley (SD) rats, aged 6–8 weeks and weighing 180 ± 20 g, were housed in cages and given ad libitum access to food and water. After one week of acclimatization, the rats were ready for experimentation. All animal experiments were approved by the Fujian Provincial Hospital Experimental Animal Ethics Committee (Fuzhou, China) and were performed under strict supervision. The study was assigned the Ethics Number: IACUC-FPH-SL-20230607[0726].

### 2.2 Preparation of DMF

Dissolve 0.5 g of sodium carboxymethyl cellulose (0.5 % CMC-Na) in 100 ml of ddH_2_O as a solvent, and configure the corresponding concentration of DMF at a gavage dose of 10 ml/kg.

### 2.3 Induction of acute kidney injury following traumatic brain injury

AKI following TBI was induced using the Feeney method of free-fall, as previously described [15]. Briefly, under aseptic conditions, rats were anesthetized with sodium pentobarbital (30 mg/kg) by intraperitoneal injection and positioned in a prone stance within a stereotactic head frame. The head skin was disinfected, turbaned, and a median incision exposed the parietal bone. A 1 cm diameter bone window was created 3.0 mm beyond the fontanel point and 2 mm to the right of the midline, preserving the integrity of the dura mater. Traumatic brain injury was induced using a free-fall craniocerebral percussion device (RWD Life Science Co., Ltd., 68009). Post-injury, the scalp was sutured. In the sham-operated group, a bone window was opened without percussion treatment.

### 2.4 Experimental groups and drug administration

The rats were randomly divided into five groups (n = 3/group): sham operation control group, model group, model + 12.5 mg/kg dimethyl fumarate (DMF) group, model + 25 mg/kg DMF group, and model + 50 mg/kg DMF group. Different treatments were administered according to the groups. The sham operation control group underwent craniotomy without brain injury induction. The model group underwent brain injury induction as described above. The other groups received different doses of the Nrf2 agonist DMF (12.5 mg/kg, 25 mg/kg, 50 mg/kg) once daily for six consecutive weeks via oral gavage following brain injury induction. At the conclusion of the administration period, all rats were humanely euthanized via intraperitoneal injection of sodium pentobarbital at a dose of 150 mg/kg. Subsequently, blood, kidney, and brain samples were collected for further analysis.

### 2.5 Assessment of renal function

Blood samples were obtained from the rats and centrifuged at 3000 rpm for 10 min to obtain serum. Serum creatinine (sCr) levels were measured using an enzyme-linked immunosorbent assay (ELISA) kit (#70127, Boibase Biodustry Co., Ltd, Shandong, China) according to the manufacturer's instructions. Neutrophil gelatinase-associated lipocalin (NGAL) levels were also measured using an ELISA kit (MM-0271R1, Meimian Industrial Co., Ltd, Jiangsu, China).

### 2.6 Hematoxylin-eosin (HE) staining

Kidney tissue sections from each group were fixed in 4 % paraformaldehyde, paraffin-embedded, and cut into 3 μm sections. These sections were then stained with hematoxylin and eosin using standard protocols. Microscopic examination under a light microscope (Olympus, Tokyo, Japan) was performed to evaluate the pathological changes and the renal injury score was calculated on the basis of the following criteria. Nine non-overlapping fields of kidney tissues were scored (under 400x magnification) according to the following criteria: glomerular necrosis, tubular degeneration, and neutrophil infiltration were scored from 0 to 4 based on the percentage of the affected area (0 points: 0 %, 1 point: >0 %–10 %, 2 points: >10 %–25 %, 3 points: >25 %–50 %, 4 points:>50 %).

### 2.7 Terminal deoxynucleotidyl transferase-mediated dUTP Nick end labeling (TUNEL) assay

Apoptosis severity in kidney tissues was assessed using the TUNEL assay (C1090, Beyotime Biotechnology, Shanghai, China). Kidney tissue sections were fixed, paraffin-embedded, and sectioned. The sections were then subjected to deparaffinization and hydration, followed by incubation with proteinase K working solution at 37 °C for 30 min. After rinsing with phosphate-buffered saline (PBS), the sections were dried and incubated with TUNEL assay solution at 37 °C for 2 h (protected from light). Subsequently, the sections were rinsed with PBS, incubated with 4′, 6-diamidino-2-phenylindole (DAPI) for 5 min (protected from light), and mounted with an antifade mounting medium after another PBS rinse. Fluorescence microscopy was used to observe the red-stained TUNEL-positive cells. The apoptosis index (AI) was calculated using the formula: AI = (number of apoptotic cells/total number of counted cells) × 100 %.

### 2.8 Determination of serum interleukin (IL)-6, IL-1β, and tumor necrosis factor (TNF)-α

Serum IL-6 levels were measured using an enzyme-linked immunosorbent assay (ELISA) kit (MM-0190R1, Meimian Industrial Co., Ltd, Jiangsu, China) according to the manufacturer's instructions. Similarly, serum IL-1β levels were measured using an ELISA kit (MM-0047R1, Meimian Industrial Co., Ltd, Jiangsu, China), and serum TNF-α levels were measured using an ELISA kit (MM-0180R1, Meimian Industrial Co., Ltd, Jiangsu, China).

### 2.9 Determination of malondialdehyde (MDA) content and superoxide dismutase (SOD) activity in kidney and brain tissues of rats

The MDA content in kidney and brain tissues was determined using an MDA assay kit (MM-0385R1, Meimian Industrial Co., Ltd, Jiangsu, China) according to the manufacturer's instructions. Similarly, the SOD activity in kidney and brain tissues was determined using a SOD assay kit (MM-0386R1, Meimian Industrial Co., Ltd, Jiangsu, China).

### 2.10 Western blotting

Protein was extracted from kidney and brain tissues. Kidney tissues (50 mg) were homogenized in 1 ml of radioimmunoprecipitation assay buffer lysis solution (C1053, Applygen Genetic Technology Co., Ltd, Beijing, China) using an automatic sample fast grinder (Tiss-12, Jingxin Industrial Development Co., Ltd., Shanghai, China). The homogenized solution was centrifuged, and the supernatant was collected for protein concentration determination using a bicinchoninic acid protein assay kit (E-BC-K318-M, Elabscience Biotechnology Co., Ltd, Wuhan, China). The protein samples (20 μg) were separated by electrophoresis and transferred to a polyvinylidene difluoride (PVDF) membrane (IPVH00010, Millipore, Bedford, MA, USA). The PVDF membrane was blocked and incubated with primary antibodies against β-Actin, Bax, BCL-2, Keap1, Nrf2, and HO-1. After washing, the membrane was incubated with corresponding secondary antibodies. The protein bands were imaged and quantitatively analyzed.

### 2.11 Statistical analysis

Data analysis was performed using SPSS 20.0 and GraphPad Prism 8.0.2. The data were expressed as mean ± standard error of the mean (SEM). The Levene test was used to assess the homogeneity of variance. Differences between the two groups were evaluated using Student's *t*-test, while differences among multiple groups were evaluated using one-way ANOVA. A p-value <0.05 was considered statistically significant.

## 3 Results

### 3.1 Detection of acute kidney injury following traumatic brain injury model

In the model group, the sCr levels were significantly higher than those in the control group (p < 0.05), indicating renal dysfunction. Similarly, the AKI marker NGAL levels were significantly higher in the model group compared to the control group (p < 0.01). Besides, the renal injury was significantly enhanced in the model group, characterized by pathologic renal morphological damage and an elevated renal injury score calculated by HE staining. These findings indicate the occurrence of renal injury in the model group, suggesting the successful establishment of the AKI following the TBI model (Fig. 1, Fig. 3B).

**Fig. 1:**
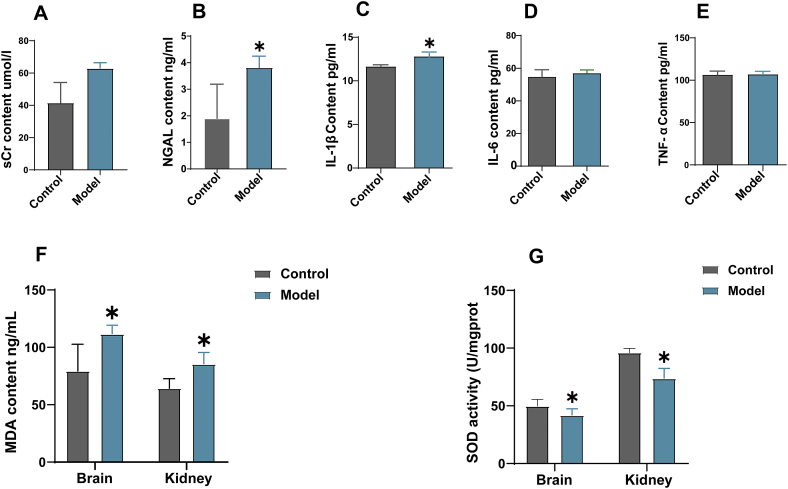
**Changes in renal function, inflammatory indexes, oxidative stress, and the regulation of the Keap1-Nrf2/HO-1 signaling pathway in rats after establishing an acute kidney injury following traumatic brain injury model**. (A) serum creatinine levels. (B) neutrophil gelatinase-associated lipocalin levels. (C) IL-1β content. (D) IL-6 content. (E) TNF-α content. (F) MDA content. (G) SOD activity. The results were presented as mean ± standard error of the mean (bar chart). *: p < 0.05.

### 3.2 Inflammatory response and oxidative stress

The levels of IL-1β were significantly increased in the model group compared to the control group (p < 0.05), while no significant differences were observed in IL-6 and TNF-α levels (Fig. 1C–E). The MDA content was significantly higher in both brain and kidney tissues of the model group compared to the control group (p < 0.01), indicating increased oxidative stress. Conversely, the SOD activity was significantly decreased in both brain and kidney tissues of the model group compared to the control group (p < 0.05), suggesting impaired antioxidant defense mechanisms (Fig. 1F and G). These results indicate the occurrence of inflammatory response and oxidative stress in the AKI following the TBI model.

### 3.3 The effect of DMF on renal function

The levels of sCr and NGAL decreased in the groups treated with DMF compared to the model group (Fig. 2A and B). Notably, a statistically significant reduction in NGAL levels was observed in both the 25 mg and 50 mg DMF-treated groups. These findings suggest a beneficial impact of DMF treatment on ameliorating renal injury.

**Fig. 2:**
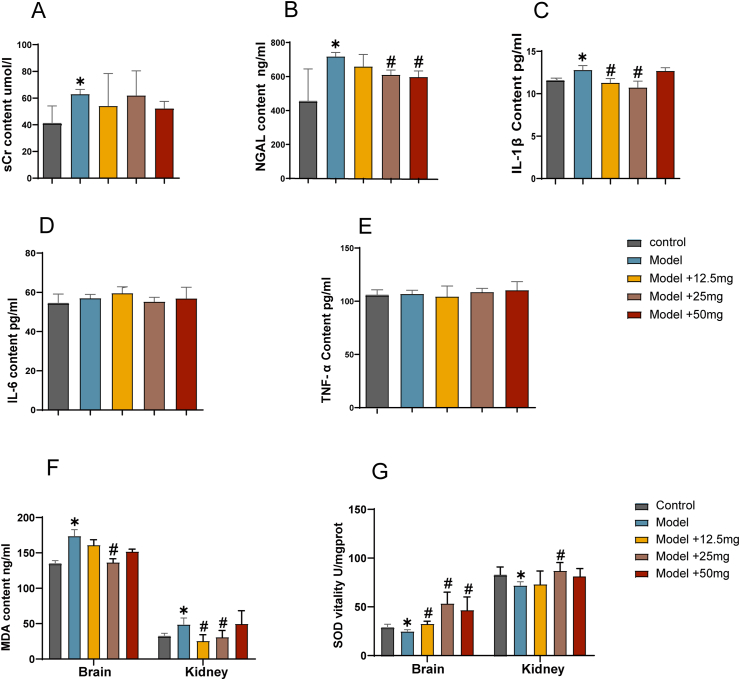
**The effect of DMF on renal function, inflammatory indexes, and oxidative stress in the kidney injury following traumatic brain injury model.** (A) serum creatinine levels. (B) neutrophil gelatinase-associated lipocalin levels. (C) IL-1β content. (D) IL-6 content. (E) TNF-α content. (F) MDA content. (G) SOD activity. The results were presented as mean ± standard error of the mean. *: p < 0.05 in comparison to control. #: p < 0.05 in comparison to the model group.

**Fig. 3:**
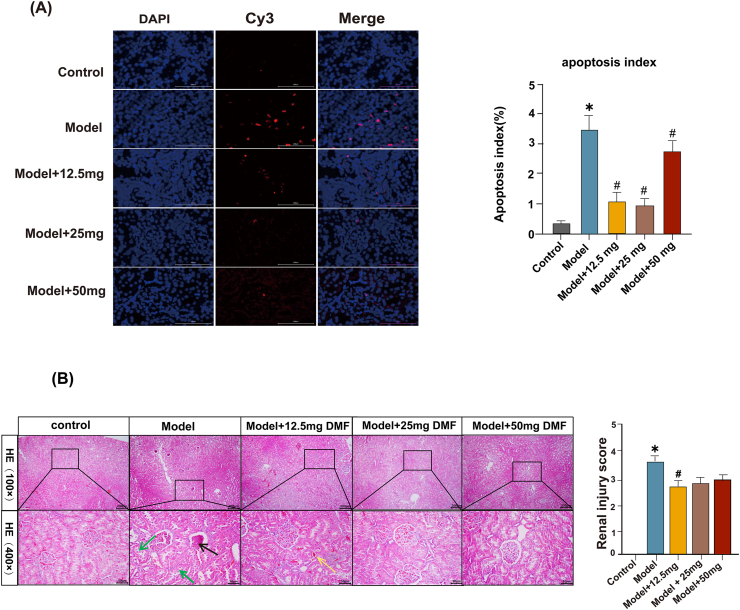
(A) TUNEL detection of apoptotic signals. DMF ameliorated apoptosis. The results were presented as mean ± standard error of the mean. *: p < 0.05 in comparison to control. #: p < 0.05 in comparison to the model group. (B) Representative HE staining in kidney tissue of rats. In the model group, glomerular atrophy (black arrows) and detachment of tubular epithelial cells (green arrows) were observed. In the 12.5 mg DMF group, improvement of glomerular lesions was observed, with haemorrhage in the tubulointerstitial space (red arrows); glomerular lesions in the 25 mg and 50 mg DMF groups tended to be normalised; and tubular lesions did not show any significant changes. *: p < 0.05 in comparison to control. #: p < 0.05 in comparison to the model group. (For interpretation of the references to colour in this figure legend, the reader is referred to the Web version of this article.)

### 3.4 The effect of DMF on inflammatory response and oxidative stress

The levels of IL-1β were significantly reduced in the DMF treatment groups compared to the model group, with the 25 mg DMF group showing the most obvious improvement (Fig. 2C). No significant differences were observed in the levels of IL-6 and TNF-α between the DMF treatment groups and the model group.

The levels of MDA were significantly decreased in the DMF treatment groups compared to the model group, while the SOD activity was significantly increased (Fig. 3F and G). However, there were no dose-dependent changes in MDA and SOD levels in brain and kidney tissues in the 50 mg DMF group compared to the 25 mg group. These findings suggest that traumatic brain injury triggers inflammation and oxidative stress, and DMF might alleviate these effects.

### 3.5 Apoptosis activity attenuated by DMF

The model group resulted in significant cell apoptosis, exhibiting an increased AI compared to the control group. Treatment with different doses of DMF resulted in attenuation of the AI with a significant reduction in the number of TUNEL-positive nuclei (Fig. 3A). Notably, the 25 mg DMF dose showed the most distinct inhibitory effect on apoptosis rather than the 50 mg DMF.

### 3.6 Renal histopathological

The model group exhibited extensive glomerular necrosis, tubular degeneration, and neutrophil infiltration, consistent with the renal function damage observed. TBI could significantly increase the renal injury scores, suggesting severe renal injury. These pathological changes were improved by DMF administration, resulting in a reduction in renal injury scores. The 12.5 mg DMF group showed the lowest renal injury scores, which was significantly lower than the other two treated groups (Fig. 3B).

### 3.7 The effect of DMF on the regulation of the Keap1-Nrf2/HO-1 signaling pathway

The model group showed elevated levels of Keap1, Nrf2, and HO-1 in both tissues. Treating with DMF increased Nrf2 and HO-1 protein levels, partially reversed the elevation of Keap1 levels, suggesting that the Keap1- Nrf2/HO-1 pathway might be related to oxidative stress. Apoptosis occurred in the model group as evidenced by the increased levels of pro-apoptotic protein Bax and the decreased levels of anti-apoptotic protein Bcl-2 in both tissues, consistent with the results of TUNEL staining. The DMF treatment partially reversed the elevation of Bax levels and restored the decrease in Bcl-2 levels (Fig. 4), and the decreased Bax/Bcl-2 levels further demonstrated that DMF could inhibit apoptosis. Whereas 50 mg DMF was not as effective as lower concentrations of DMF in increasing Nrf2 and HO-1 levels in brain tissue, and there was no significant dose-effect relationship for changes in the levels of Bax, Bcl-2, and Keap1 with increasing doses of DMF.

**Fig. 4:**
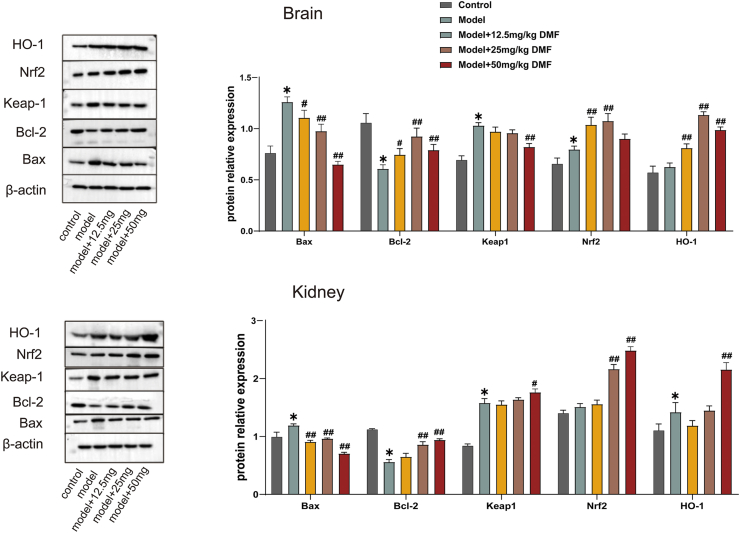
**The effect of DMF on protein expression of Bax, Keap1, Nrf2, HO-1, and Bcl-2 in rats' kidney and brain tissue.** The results were presented as mean ± standard error of the mean. *: p < 0.05 in comparison to control. #: p < 0.05 in comparison to the model group. ##: p < 0.01 in comparison to the model group. The non-adjusted images have been uploaded as supplementary material.

## 4 Discussion

The main findings of this study were: 1) severe kidney damage was observed after TBI, which was associated with increased oxidative stress, inflammation, and apoptosis; 2) alterations in the pathways regulating oxidative stress, including Nrf2, HO-1, Bax/Bcl-2, and Keap1, were observed; 3) DMF treatment inhibited oxidative stress of AKI after TBI by upregulating the Keap1-Nrf2/HO-1 signaling pathway, thereby attenuating renal function impairment and apoptosis.

TBI can lead to AKI with serious consequences. The treatment of AKI not only consumes a large amount of medical resources and increases the social burden but also causes great distress to families, particularly the patients themselves. Therefore, it is essential to investigate the molecular mechanisms underlying AKI following TBI and explore effective preventive and therapeutic measures. Activation of oxidative stress is considered a potential pathological mechanism, and the Keap1-Nrf2/HO-1 signaling pathway is an important signaling pathway against oxidative stress. Nevertheless, studies on whether the Nrf2 agonist DMF can exert renoprotection by reducing oxidative stress after TBI are scarce. Thus, we proposed that TBI induces oxidative stress and AKI by regulating the Keap1-Nrf2/HO-1 signaling pathway, whereas up-regulation of this pathway could ameliorate this phenomenon.

The body rapidly generates a large number of free radicals through various pathways after TBI, damaging the body by attacking biological macromolecules such as lipids, proteins, and nucleic acids [16]. The increase in MDA and decrease in SOD directly reflect the degree of oxidative stress, and Loganin could alleviate AKI in septic mice by decreasing MDA levels and increasing SOD activity [17]. Our study presented that TBI rats had elevated sCr, NGAL, IL-1β, and MDA levels and decreased SOD levels, accompanied by upregulation of Keap1, Nrf2, HO-1 and Bax/Bcl-2. Elevated IL-1β levels indicated the expression of pro-inflammatory cytokines in response to inflammation, and elevated Bax/Bcl-2 ratio and TUNEL staining results showed apoptosis. In summary, TBI caused oxidative stress, inflammation, and apoptosis via the Keap1-Nrf2/HO-1 pathway, exacerbating kidney injury. Similar to our findings, Kontos et al. found increased oxidative stress activation within a short period after TBI in animal models of hydraulic shock injury [18], leading to a decrease in cerebrovascular autoregulation and pathological changes in arterial endothelial cells and smooth muscle cells, which could be improved by free radical scavengers. Previous studies also noted that nitric oxide synthetase (NOS) catalyzes the production of large amounts of NO, and all free radicals derived from ONOO^−^ generated from its reaction with O_2_^−^ cause oxidative stress damage to biological macromolecules [19]. 3-Nitrotyrosine (3-NT), formed by tyrosine nitration, is one of the common indicators of peroxynitrite-induced oxidative stress. Animal models of TBI had significantly higher levels of 3-NT [20], while administration of NOS inhibitors reduced the accumulation of 3-NT and attenuated brain injury [21]. Based on these findings, it suggests that TBI can induce oxidative stress and cause damage to distant organs.

In addition, lipid peroxide (LPO) is one of the biological indicators of aging. The oxidative stress damage occurring in the body under pathological conditions will generate more LPO, which corresponds to an acceleration of the aging process. What's more, lipid peroxidation can form reactive carbonyl species, mainly some unsaturated aldehydes such as 4-hydroxynonenal, acrolein, MDA, and these reactive carbonyl molecules are able to carbonylate proteins, thus causing abnormalities in protein structure and function [22]. Animal experiments have demonstrated a significant increase in LPO levels after TBI, as well as a significant increase in carbonyl protein levels [23]. Therefore, alleviation of oxidative stress damage is necessary for the control of secondary diseases after TBI.

In our study, the model group had an elevated Bax/Bcl-2 ratio and an increased apoptotic index with the activation of oxidative stress after TBI, confirming that oxidative stress induces apoptosis. Studies have shown that the oxidative stress-mediated apoptotic signaling pathway can be divided into the mitochondrial and mitogen-activated protein kinase (MAPK) pathways. The former refers to the direct activation of mitochondria by reactive oxygen species (ROS) during oxidative stress, which upregulates the Bax/Bcl-2 ratio. This pathway involves enhanced mitochondrial membrane permeability, which in turn leads to apoptosis via caspase-dependent or caspase-independent pathways [24]. It has been observed that mitochondrial apoptotic pathway activation played a crucial role in increasing the Bax/Bcl-2 ratio and triggering oxidative stress-induced apoptosis after abivertinib intervention [25]. This is most likely to be achieved through the mitochondrial pathway and still needs to be confirmed by further study. On the other hand, in the MAPK pathway, ROS promotes apoptosis by activating caspase through the phosphorylation of JNK and p38 [26]. Recently, there have been some new findings, such as HAX-1 mutation (Q190X) or deficiency [27] and C-X-C motif chemokine ligand 1 (CXCL1) [28], may also be related to oxidative stress-induced apoptosis. In addition to promoting apoptosis [29], ROS are important for the production of inflammatory factors. Increased ROS contribute to the phosphorylation and degradation of IκB, enhance NF-κB signaling, and activate inflammatory mechanisms [30,31]. Oxidative stress and inflammatory responses are mutually reinforcing in the development of kidney injury. Oxidative stress can contribute to the development of inflammation, and the cellular damage caused by inflammation further aggravates oxidative stress.

So far, there is increasing evidence that oxidative stress plays an essential role in the development of AKI. Despite some progress, there still lacks available treatments. Therefore, exploring new drugs or therapeutic targets for treatment is of great value and significance. DMF has demonstrated its efficacy in various human diseases. It has been shown to reduce pulmonary fibrosis by inhibiting macrophage TGF-β and ROS production in idiopathic pulmonary fibrosis [32], mitigate the disease by reducing fibrotic and inflammatory processes in systemic sclerosis [33], and also exhibit brain-protective effects through autophagy activation and modulation of neuroinflammation in Parkinson's disease [34]. However, it remains unclear whether DMF has therapeutic potential for AKI following TBI. In this study, we investigated the effectiveness of DMF in treating AKI after TBI. Our findings indicate that DMF treatment increased SOD activity and reduced MDA content in the brain and kidney tissues of rats with AKI after TBI, effectively inhibiting oxidative stress.

The Keap1-Nrf2/HO-1 signaling pathway has been confirmed as one of the most noteworthy anti-oxidative stress pathways in previous studies [35,36]. Usually, Nrf2 is negatively regulated by Keap1 and maintained at low cellular concentrations. Once the cell is stimulated by damage, Nrf2 will overcome the inhibition of Keap1 and translocate to the nucleus, subsequently activating genes downstream of the pathway, including HO-1. kidney injury by activating Nrf2, whereas its protective effect was abolished in Nrf2 KO mice, revealing the reno-protective effect is exerted through the Nrf2 target [37]. We found that Nrf2 and HO-1 levels were elevated in the model group compared to the control group, which was the consequence of oxidative stress stimulation. As expected, Nrf2 levels were further elevated after DMF administration, and downstream HO-1 levels were also further elevated, which acted as an anti-oxidative stressor and counteracted the elevated Bax/Bcl-2 levels and increased apoptosis due to oxidative stress. Taken together, these results confirm that DMF exhibited a reno-protective effect in TBI rats.

The nervous system is rich in iron, and the iron-rich brain tissue was found to have a more severe oxidative damage effect in animal models of brain injury [38]. In the presence of hydrogen peroxide, Fe^2+^ can generate ·OH through the Fenton reaction, leading to oxidative stress damage to the body. In addition, trauma can cause bleeding, a condition in which the clot's hemoglobin can directly stimulate the production of free radicals and cause oxidative stress damage to the neurological system by releasing iron ions [39]. To date, compelling evidence suggests that HO-1 is a key protein in the onset of ferroptosis, and many drugs, including Celastrol [40], Quercetin [41], and Salvianolic acid B [42], inhibit ferroptosis by activating Nrf2/HO-1 signaling. The results of our study suggest that DMF inhibits oxidative stress-induced AKI after TBI by upregulating the Keap1-Nrf2/HO-1 signaling pathway.

However, this study still has some limitations. First, only animal models have been studied; more comprehensive in vitro and clinical studies are still needed. Second, we could not identify the optimal dosage of DMF since this was not the original intention of our research design.

Taken together, our findings reveal DMF can attenuate oxidative stress-induced AKI after TBI by activating Keap1-Nrf2/HO-1 signaling pathway, thus supporting Nrf2 as a therapeutic target for AKI after TBI, as well as further development of DMF as a promising therapeutic agent for AKI after TBI.

## Ethics statement

The study received approval from the Fujian Provincial Hospital Experimental Animal Ethics Committee (Fuzhou, China), with the ethics approval number IACUC-FPH-SL-20230607[0726].

## Funding

This work was supported by the 10.13039/501100003392Fujian Provincial Natural Science Fund Project (Grant Number: 2021J01369, 2023J011159), the Fujian Provincial Health Technology Project (Grant Number: 2021GGA002), and the Youth Top Talent Project of Fujian Provincial Foal Eagle Program.

The funders had no role in study design, data collection and analysis, decision to publish, or preparation of the manuscript.

## Data availability statement

The data used to support the findings of the present study are available from the corresponding author upon request.

## CRediT authorship contribution statement

**Mei-zhu Gao:** Writing – original draft, Investigation, Formal analysis, Data curation. **Jing-yi Zeng:** Investigation, Formal analysis, Data curation, Conceptualization. **Xue-jing Chen:** Resources, Methodology. **Lan Shi:** Validation, Methodology. **Fu-yuan Hong:** Resources, Methodology. **Miao Lin:** Methodology, Investigation. **Jie-wei Luo:** Writing – review & editing, Supervision, Investigation, Funding acquisition. **Han Chen:** Writing – review & editing, Supervision, Project administration, Funding acquisition, Formal analysis.

## Declaration of competing interest

The authors declare that they have no known competing financial interests or personal relationships that could have appeared to influence the work reported in this paper.

## Figures and Tables

**figs1:**
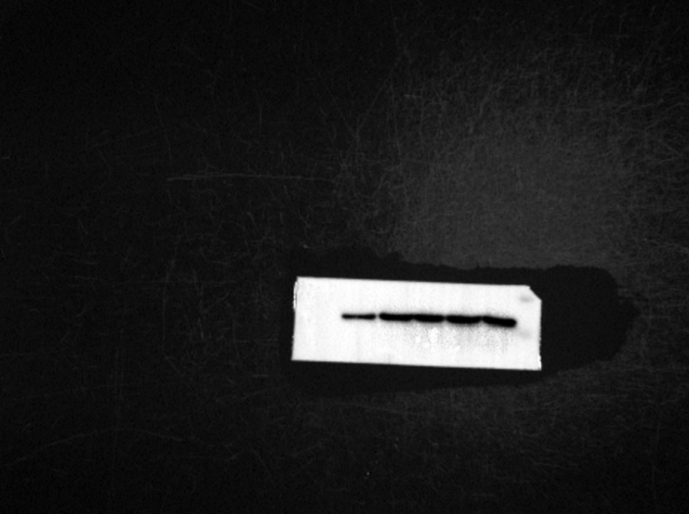


**figs2:**
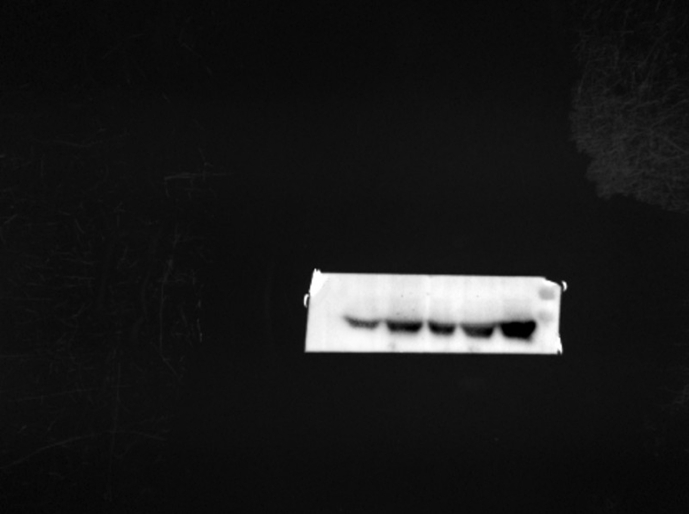


**figs3:**
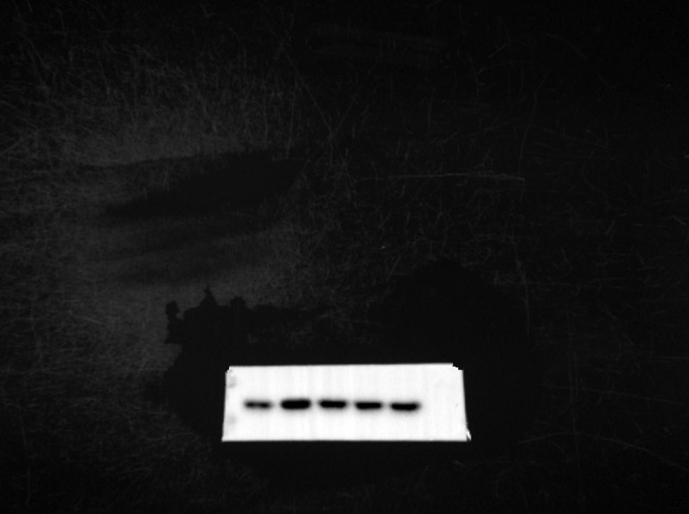


**figs4:**
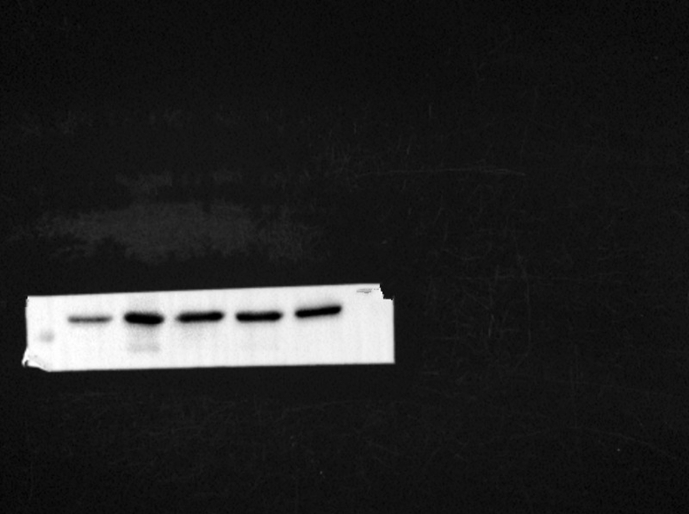


**figs5:**
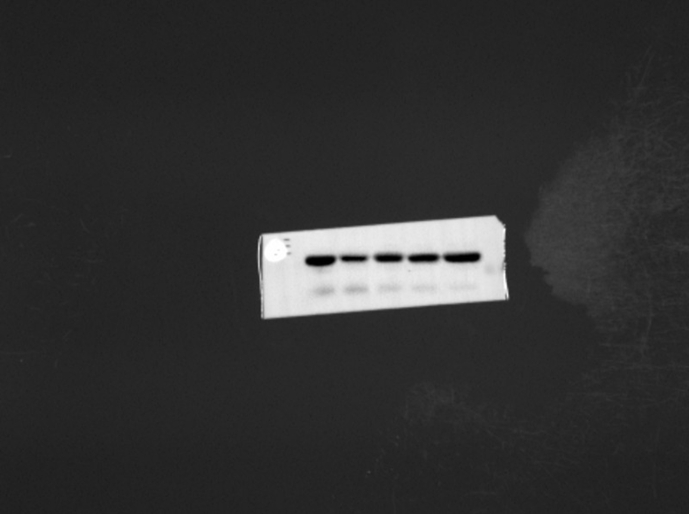


**figs6:**
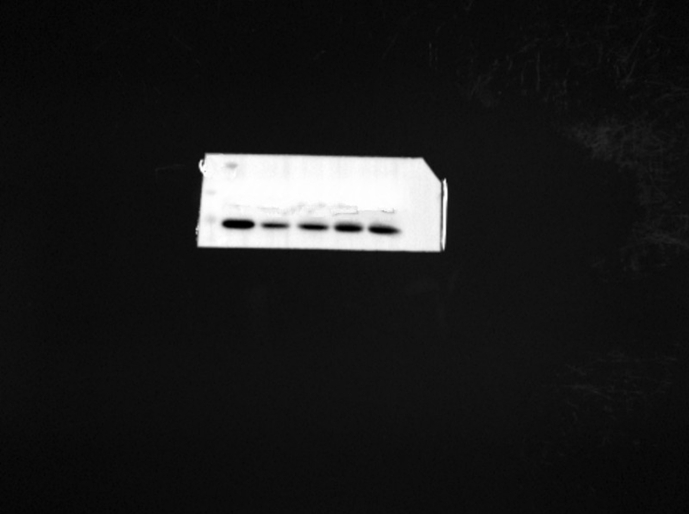


**figs7:**
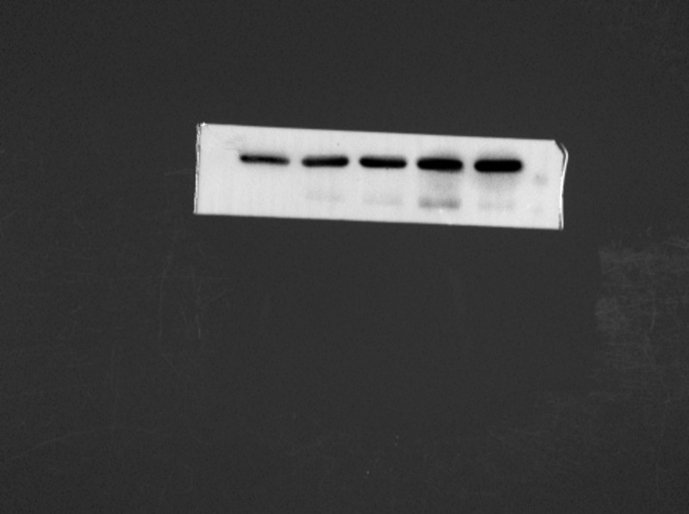


**figs8:**
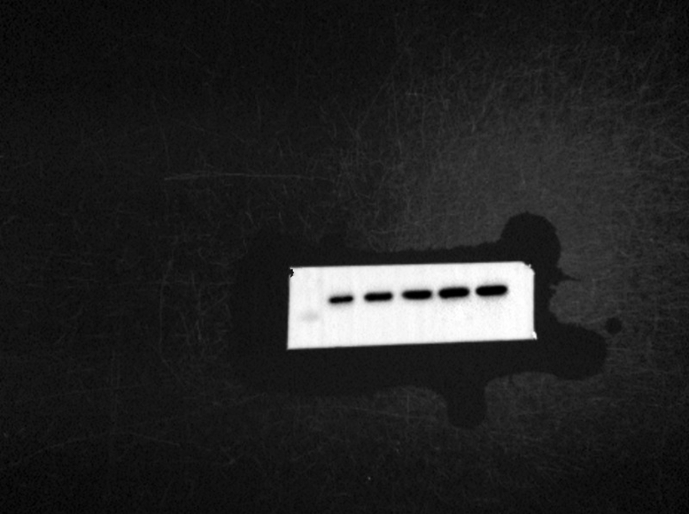


**figs9:**
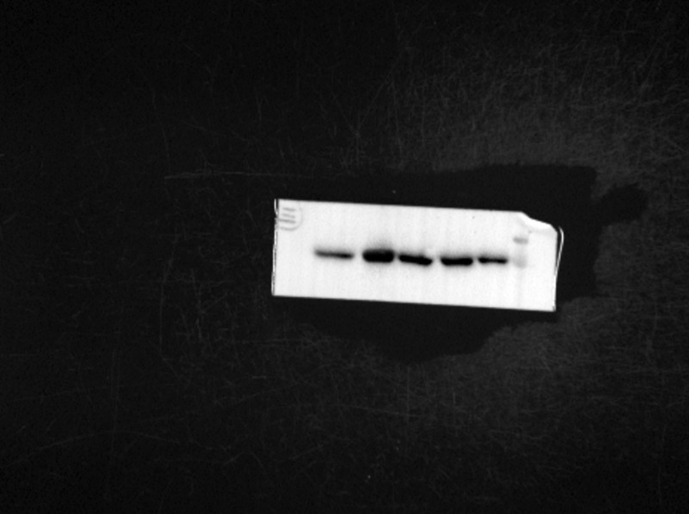


**figs10:**
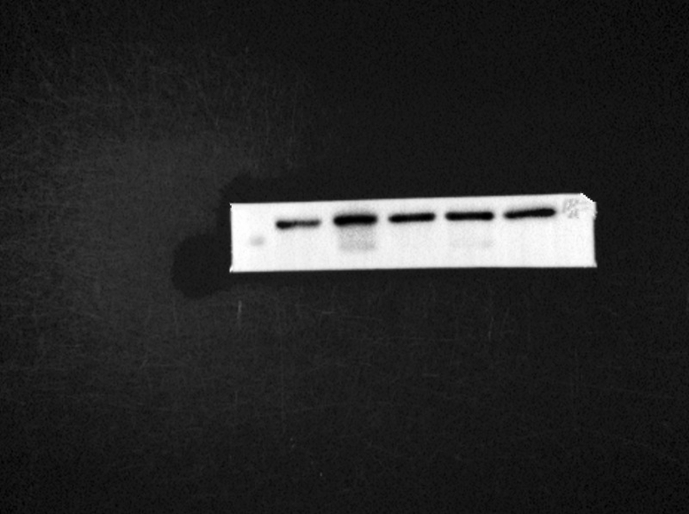


**figs11:**
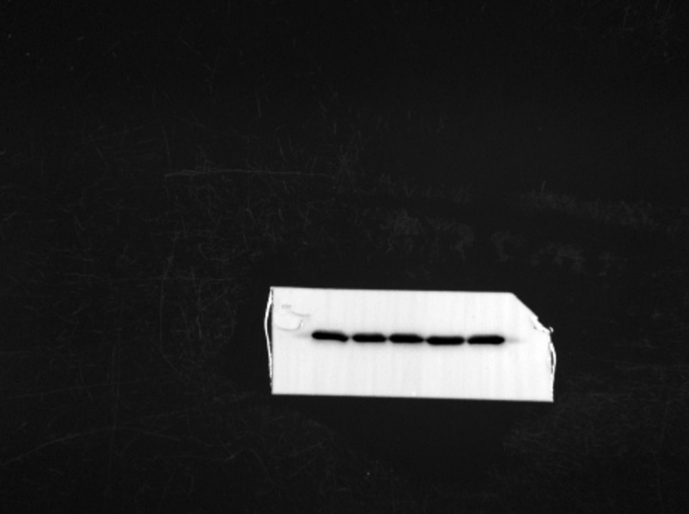


**figs12:**